# Peliosis hepatis associated with follicular lymphoma with a rise in vascular endothelial growth factor and anaemia of inflammation

**DOI:** 10.3332/ecancer.2018.882

**Published:** 2018-11-20

**Authors:** Emilia Pardal de la Mano, Guillermo Martín-Sánchez, Rosa López López, M Angeles Fernández Galán, Sergio Trinidad Ríos, M José Morán Jiménez, J María García Ruiz de Morales, M Antonia Crespo Santos, Guillermo Martín Núñez

**Affiliations:** 1 Haematology Department, Virgen del Puerto Hospital, Paraje de Valcorchero s/n, 10600 Plasencia, Spain; 2 Haematology Department, University Hospital Marqués de Valdecilla, Avd Valdecilla 25, 39008 Santander, Spain; 3 Radiodiagnosis Department, Virgen del Puerto Hospital, Paraje de Valcorchero s/n, 10600 Plasencia, Spain; 4 Research Institute, University Hospital 12 de Octubre, Avd Córdoba s/n, 28041 Madrid, Spain; 5 Immunology Service, University Hospital of León, Altos de Navas s/n, 24071 León, Spain; 6 Department of Anatomical Pathology, Virgen del Puerto Hospital, Paraje de Valcorchero s/n, 10600 Plasencia, Spain; 7 Haematology and Haemotherapy Department, Virgen del Puerto Hospital, Paraje de Valcorchero s/n, 10600 Plasencia, Spain

**Keywords:** follicular lymphoma, peliosis, vascular endothelial growth factor, anaemia of inflammation, hepcidin, interleukin 6

## Abstract

Follicular lymphoma does not usually present with associated paraneoplastic syndromes. We describe the case of a patient diagnosed with follicular lymphoma when investigating anaemia of chronic disease/inflammation and who, during her clinical course, developed peliosis hepatis. We have been able to confirm the similarity between the symptoms, the tumour’s biology, the anaemia and peliosis, with the behaviour of endothelial growth factor, interleukins and iron metabolism disorders, which were normalised with treatment. To date, we have found no cases where peliosis has been described in this type of lymphoma.

## Introduction

Peliosis is a rare vascular anomaly characterised by the presence of sinusoidal dilatation and multiple blood-filled lacunar spaces. It is often located in the liver but also in the spleen, lung and other organs [1]. It is observed in patients with different diseases, especially those with an important inflammatory component: tuberculosis, carcinomatosis, haematological tumours (Hodgkin lymphoma [2], Castleman’s disease), HIV infection and bacteria—*Bartonella henselae—*and anabolic treatments, in some kidney transplant recipients, etc. In general, it is usually asymptomatic, discovered when studying abnormalities in blood tests or liver imaging, although there have been reports of severe and recurrent peritoneal haemorrhages and liver failure [3]. Its etiopathogenesis is unclear. It has been related to endothelial damage caused by toxic and infectious agents, and the possibility that these agents increase the production of vascular endothelial growth factor (VEGF) [4], which is a potent angiogenic factor. On the other hand, an anaemia of chronic disease or inflammation is usually associated with tumours, especially those that secrete cytokines by different mechanisms, especially IL6, such as renal clear cell carcinoma [5], and in some lymphomas with a large inflammatory component such as Hodgkin lymphoma, some T-cell lymphomas and in Castleman’s disease [6]. In the case we present, we have been able to observe both processes, chronic anaemia and peliosis associated with lymphoma and its response to treatment.

## Clinical case

**Figure 1.**
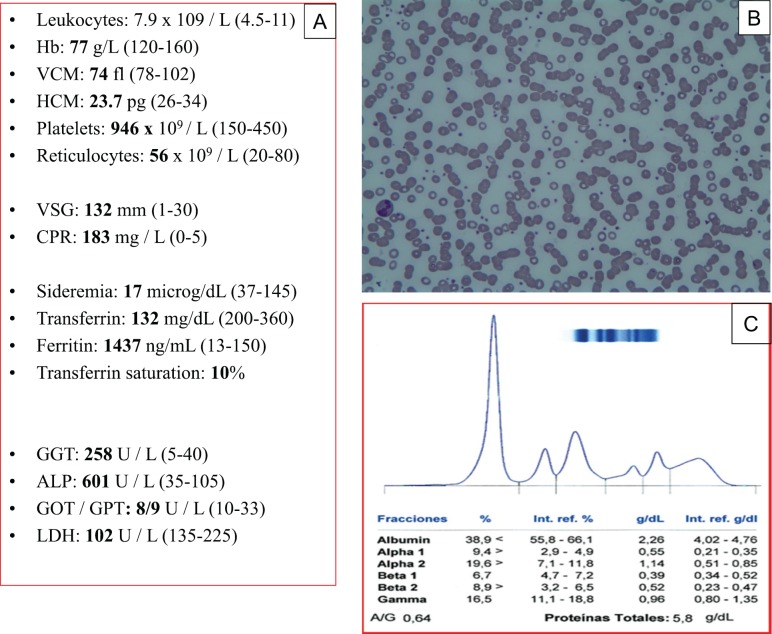
Analytical data: (A) Hyporegenerative microcytic anaemia, abnormality in iron metabolism (Fe), elevation of acute phase reactants and liver enzyme abnormality. (B) Morphology of peripheral blood with hypochromia, intense rouleaux and thrombocytosis. (C) Proteinogram with hypoalbuminemia and hypergammaglobulinemia.

A 68-year-old woman referred to the haematology outpatients department of our centre (Virgen del Puerto Hospital, Plasencia) in August 2015 with progressive anaemia detected 4 months earlier with asthenia, anorexia, profuse sweating and a weight loss of 6 kg. She was treated with dicumarinics for atrial fibrillation with no relevant history. On physical examination: performance status 1, skin pallor and a small axillary lymph node. The analytical and peripheral blood morphology data are shown in Figure 1.

Imaging studies: Abdominal ultrasound: liver with a slight increase in overall size, with homogenous parenchyma of normal echogenicity and with no focal lesions. Homogeneous splenomegaly of 14 cm. Portal vein slightly enlarged. Chest X-ray: no changes. Computerised tomography (CT scan): absence of mediastinal adenopathies. Liver slightly increased in size with heterogeneous densitometry without demonstrating focal lesions. Spleen at the upper limit of normality. Small retroperitoneal adenopathies measuring 11 mm. Mammogram: normal.

Bone marrow aspiration: reactive and with no morphological evidence of tumour infiltration. Increased iron deposits, no sideroblasts.

Other studies: gastroscopy and colonoscopy: normal. Core-needle biopsy-aspiration of axillary ganglion cyst: could not be assessed.

The patient was periodically checked in the outpatients clinic and 2 months after the start of the investigation, and in view of the persistence of asthenia and anaemia, we decided to perform a splenectomy, removing a spleen measuring 12 × 11.5 × 6.5 cm and weighing 317 g. Five nodular formations were detected, the largest measuring 0.5 cm in white pulp, composed of germinal centre-type cells compatible with non-Hodgkin follicular lymphoma (NHFL). Therapeutic abstention was decided given the good tolerance to anaemia and the biological characteristics of the lymphoma.

**Figure 2.**
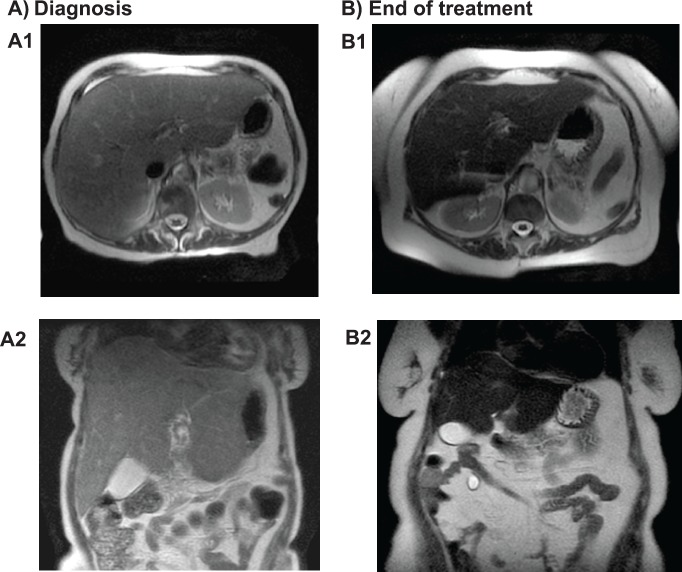
Abdominal magnetic nuclear resonance: (A) Axial (A1) and coronal (A2) view during diagnosis: increased liver size, with finely heterogeneous parenchyma and no focal lesions. (B) Axial (B1) and coronal (B2) view after treatment: liver size within normal limits with homogeneous parenchyma.

**Figure 3.**
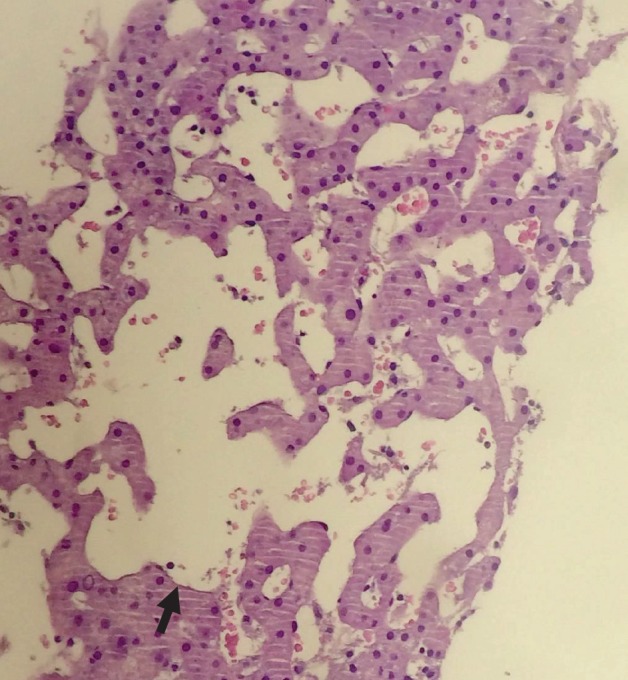
Peliosis hepatis. Cystic spaces with no endothelial lining (arrow). HE × 10.

At 6 months, the anaemia progressed and hepatomegaly was detected 6 cm below the costal margin. During magnetic nuclear resonance (MNR), we observed severe hepatomegaly suggestive of infiltration, with increased retroperitoneal adenopathies and compromise of the right renal excretory duct (Figure 2). Positron emission tomography/computerised axial tomography (PET/CT scans) were performed as a staging evaluation: multiple hypermetabolic ganglion lesions in the retroperitoneum (SUVmax 7) in blocks, the largest measuring 2.4 × 3.0 cm, the hepatomegaly being better evaluated by other techniques such as the MNR. The liver biopsy shows slight portal fibrosis, marked dilation of the sinusoids that appear filled with blood material and with a tendency to form cystic cavities, compatible with peliosis hepatis (Figure 3).

After confirming the progression of NHFL with clinical deterioration, treatment was started with immuno-chemotherapy type R-CHOP with good tolerance (six cycles of R-CHOP with maintenance rituximab every 2 months for 2 years). After the third cycle, an improvement in anaemia and of the inflammatory patterns, including those of iron metabolism, was observed. In the evaluation after six cycles of R-CHOP, no foci of uptake were detected on the PET/CT scan, no adenopathies were observed and the liver was normal in size and appearance.

Serial analytical determinations of hepcidin and cytokines (IL-6, VEGF) were performed from the moment of diagnosis, at the start of treatment, and before and at the end of each cycle of chemotherapy. Table 1 shows the evolution of these parameters, demonstrating their decrease from the first cycle, highlighting the decrease in IL-6 in parallel to the decrease in hepcidin and VEGF. Likewise, the decrease in liver enzymes occurred in parallel with the reduction in hepatomegaly. Radiological data (CT and MRI scans) confirmed this regression of peliosis (Figure 2). Currently, the patient has completed 2 years of maintenance therapy with a good general condition and complete remission of her disease.

## Discussion

Anaemia of chronic disease (ACD) is a consequence of an inflammatory state associated with different situations (infectious, tumoural and inflammatory) in which there is an activation of the mononuclear phagocyte system (MPS) with an increase in the production of proinflammatory cytokines, including IL-6 (Figure 3). IL-6 is a pleiotropic cytokine, which stimulates different cell populations, as it is a potent inducer of acute phase reactants synthesis (CRP, fibrinogen), hepcidin and VEGF by fibroblasts [7] in the liver. The state of hyperhepcidinemia generated by IL-6 blocks iron in the MPS, causing a state of hypoferremia that, together with other mechanisms, develops ACD [8]. In this case, NHFL was discovered that, in spite of the low initial tumour mass, scarcely infiltrated the spleen, showed an intense increase of cytokines, especially of IL-6, VEGF and hepcidin, and a blockage of iron in the MPS, which normalised after treatment.

**Figure 4.**
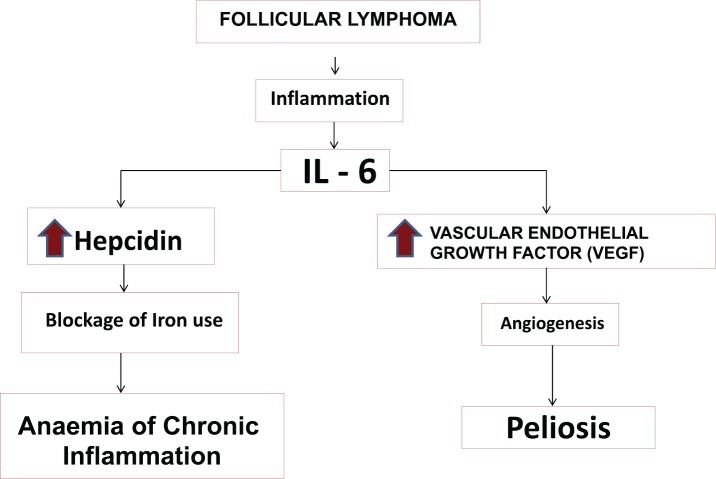
Pathogenic hypothesis of peliosis and anaemia of chronic disease in the context of follicular lymphoma in this case.

Angiogenesis is a crucial process in the growth and development of metastasis of many types of tumours, including non-Hodgkin lymphomas, with VEGF being the main regulator thereof, which is why it is considered a useful biomarker for establishing prognosis in these tumours [9]. Its increase has been observed and associated with some cases of peliosis [4]. In our patient, VEGF was raised as well as IL6 and CRP, parameters that decreased simultaneously; their decrease was observed while iron metabolism normalised, anaemia was corrected and the peliosis disappeared. The peliosis in our patient could have been caused by the elevation of VEGF as a consequence of an increase in IL-6 (Figure 4), within the inflammatory context caused by tumour. The normalisation of VEGF and hepcidin, in parallel to IL6 after treatment, and the tendency of ALP and GGT to normalise, in addition to the radiological abnormalities attributable to vascular growth in the liver; are further evidence supporting the influence of the angiogenic effect on the development of peliosis in this patient. Currently, in addition to the data described in the table, the patient maintains a marked hypogammaglobulinemia without clinical repercussion, probably related to immunochemotherapy (rituximab) she received [10].

## Conclusion

In the bibliography reviewed (PubMed), we have found no cases describing peliosis hepatis in NHFL as a paraneoplastic syndrome. We have been able to establish a possible aetiological and evolutionary similarity of some of its signs and symptoms with the secretion of different cytokines (IL-6, VEGF) and with hepcidin. We also show that in the face of anaemia of inflammation, given the processes with which it is associated, its aetiological study is very important since in many cases it can be the first step in the diagnosis of a treatable disease, as in this case. The best treatment for anaemia of inflammation is eliminating the triggering cause. In our case, the regression of lymphoma normalised iron metabolism and the peliosis disappeared.

## List of Abbreviations

In order of appearance:

VEGF: Vascular endothelial growth factor
IL: Interleukins
CT: Computerised tomography
NHFL: Non-Hodgkin follicular lymphoma
MNR: Magnetic nuclear resonance
PET/CT: Positron emission tomography/computerised axial tomography
SUVmax: Maximum standardised uptake value
R-CHOP: Rituximab, cyclophosphamide, adriamycin, vincristine and prednisone
ACD: Anaemia of chronic disease
MPS: Mononuclear phagocyte system
CRP: C reactive protein
ALP: Alkaline phosphatase
GGT: Gamma-glutamyl transpeptidase

## Conflicts of interest

The authors declare that they have no conflicts of interest and did not receive funding for the publication of this case/manuscript.

## Author contributions

Concept of study and design, data analysis, drafting and critical review of the manuscript: *E Pardal de la Mano, G Martín-Sánchez, R López López, MA Fernández Galán, G Martín Núñez.*

Determination of analytical and image tests: *S Trinidad Ríos, MJ Morán Jiménez, JM García Ruiz de Morales, MA Crespo Santos.*

The number of authors is justified by the complexity of the case and the need for other departments and hospitals to complete the investigation.

## Figures and Tables

**Table 1.** Evolution of analytical parameters during diagnosis and treatment.

Evolution	Hb g/L (120–160)	CRP mg/L (0–5)	ALP U/L (35–105)	GGT U/L (5–40)	Iron μg/L (37–145)	TRF mg/dL (200–360)	Ferritin ng/mL (13–150)	Hepcidine ng/mL (2–26)	VEGF (pg/mL) (<128)	IL-6 (pg/mL) (<7)
**(Day 0) 1st cycle**	64	229	578	249	19	149	1542	98	219	123
**(Day + 21) 2nd cycle**	113	1	178	126	89	259	996	50	3	6
**(Day + 42) 3rd cycle**	102	9	142	171	102	226	1293	46	3	6
**(Day + 63) 4th cycle**	104	5	186	179	77	273	1277	11	3	6
**(Day + 84) 5th cycle**	102	9		173	81	265	1590	24	3	6
**(Day + 150) Evaluation after 6th cycle/End of treatment**	100	5	244	281	82		812	52	3	6
**End Rituximab Therapy (2 years)**	115	3	158	320	72	265	495	53	3	6
**1 year after end of therpy treatment**	116	3	79	169	57	243	252			

Abbreviations: Hb = haemoglobin, CRP = C-reactive protein, ALP = alkaline phosphatase, GGT = gamma-glutamyl transaminase, TRF = transferrin, VEGF = vascular endothelial growth factor, IL = interleukin
